# Adapted Massed Exposure Therapy Enhanced with MDMA for PTSD (aMETEMP): A randomized, double-masked protocol adapted for Veterans Affairs healthcare system

**DOI:** 10.1016/j.conctc.2026.101705

**Published:** 2026-10-27

**Authors:** L.A. Morland, B. Chargin, B. Rothbaum, J.L. Maples-Keller, T. Wachsman, S.B. Norman, S.N. Adams, D. Perivoliotis, A. Alam, C.S. Stauffer, A.C. Angkaw, V. Marquez, J. Woolley, J.M. Kopelman, P.P. Schnurr

**Affiliations:** a Department of Veterans Affairs National Center for PTSD Women's Health Sciences Division, 150 South Huntington Street, Boston, MA, 02130, USA; b Veterans Affairs San Diego Healthcare System, 3350 La Jolla Village Drive, San Diego, CA, 92161, USA; c Department of Psychiatry, University of California San Diego School of Medicine, 9500 Gilman Drive, La Jolla, CA, 92093, USA; d Department of Psychiatry and Behavioral Sciences, Emory University School of Medicine, 12 Executive Park Drive, Atlanta, GA, 30329, USA; e San Diego State University/University of California San Diego Joint Doctoral Program in Clinical Psychology, 6363 Alvarado Court, San Diego, CA, 92120, USA; f Department of Psychiatry, Oregon Health & Science University, 3181 SW Sam Jackson Park Rd, Portland, OR, 97239, USA; g Veterans Affairs Portland Health Care System, 3710 SW US Veterans Hospital Rd, Portland, OR, 97239, USA; h Translational Psychedelic Research Program, Department of Psychiatry and Behavioral Sciences, Weil Center for Neuroscience, University of California, San Francisco, 2130 Fulton Street, San Francisco, CA, 94117, USA; i Veterans Affairs San Francisco Healthcare System, 4150 Clement Street, San Francisco, CA, 94121, USA; j National Center for PTSD Executive Division, VA Medical Center, 215 N. Main Street, White River Junction, VT, 05009, USA; k Department of Psychiatry, Geisel School of Medicine at Dartmouth, 1 Rope Ferry Road, Hanover, NH, 03755, USA

**Keywords:** Veteran, PTSD, MDMA, Massed prolonged exposure

## Abstract

Posttraumatic stress disorder (PTSD) remains prevalent and disabling, especially among veterans, with a substantial proportion failing to achieve remission following first-line evidence-based trauma-focused psychotherapies. 3,4-Methylenedioxymethamphetamine (MDMA) is hypothesized to enhance psychotherapy outcomes through effects on fear extinction, cognitive flexibility, and emotional engagement, and is being studied as a promising augmentation strategy for PTSD psychotherapy. There is limited research combining MDMA with evidence-based PTSD treatment. MDMA-assisted psychotherapy trials to date have also been limited by lack of an active comparator and functional unmasking. We describe the methodology of an ongoing randomized, double-masked, pilot trial evaluating massed prolonged exposure (PE) therapy enhanced with MDMA for PTSD (aMETEMP), adapted for a U.S. Veterans Affairs outpatient setting. Up to 20 veterans with PTSD in the VA will receive a 6-8hr in-person MDMA dosing session (randomized dose 35 mg - 125 mg) integrated with a 3-week massed exposure protocol. The remaining visits are virtual and include PE sessions and sessions to prepare for and process the MDMA session (28-30hrs total over 11 visits), with the use of telehealth intended to improve access and ease patient burden. After treating the first 4 participants, who were fully informed of the trial design, we switched to using an incomplete disclosure strategy (not disclosing the number of treatment arms or specific possible doses) to further improve masking. Primary outcomes include PTSD severity using the Clinician-Administered PTSD Scale for DSM-5, safety, feasibility, and masking success. Findings are intended to provide preliminary descriptive information regarding feasibility, safety/tolerability, masking integrity, and symptom-change signals for aMETEMP with incomplete disclosure in a large outpatient healthcare system.

**ClinicalTrials.gov Identifier:**

NCT06117306.

## Highlights

• Increasing interest in enhancing evidence-based psychotherapy for PTSD with MDMA.
• Scalable protocol of MDMA enhanced exposure therapy for PTSD within the VA.
• Incomplete disclosure and active comparator to address functional unmasking.
• Evaluate efficacy, safety, and feasibility of MDMA enhanced prolonged exposure.

Posttraumatic stress disorder (PTSD) is a chronic and debilitating psychiatric disorder, with estimated global lifetime rates of 3.9% in civilians [1], approximately 7% in U.S. veterans overall [2], and up to 20% in those exposed to combat [3]. Left untreated, PTSD is associated with a cascade of challenges including increased psychiatric and medical comorbidities, interpersonal and occupational difficulties, suicide risk, and healthcare utilization [2], [4], [5], [6]. While PTSD is considered a treatable condition, and trauma-focused psychotherapies recommended by clinical practice guidelines as first-line treatments are effective [7], [8], a substantial proportion of patients fail to achieve remission or discontinue treatment prematurely [9] and military populations demonstrate worse post-treatment PTSD remission rates compared to civilians [10]. Therefore, novel augmentation strategies that can enhance first-line PTSD treatments are needed. Ideally, these treatment models would be accessible and implemented in large integrated health settings such as the Veterans Affairs (VA) healthcare system.

There is tremendous interest in the potential of 3,4-methylenedioxymethamphetamine (MDMA) to augment PTSD psychotherapy. Pharmacologically, MDMA promotes release, inhibits reuptake, and prolongs synaptic activity of serotonin, norepinephrine, and dopamine, with the serotonergic effects believed to account for most of MDMA's subjective effects, while norepinephrine and dopamine effects are believed to account for most of MDMA's physiological effects [11], [12]. The monoamine-releasing effects of MDMA lead to the release of downstream signaling molecules thought to be important for neuroplasticity, such as brain-derived neurotrophic factor, as well as hormones such as oxytocin and cortisol, that affect the emotional memory circuits relevant to PTSD and fear extinction [13]. Extinction learning has been found to be better retained when enhanced with MDMA [14]. Psychologically, MDMA has been reported to enhance feelings of trust and emotional openness and may increase capacity to engage with painful trauma-related material with greater emotional tolerance, all of which are important factors for facilitating trauma processing [15], [16]. Further, preclinical research suggests MDMA may induce a 2-week “neuroplastic window” of heightened learning and emotional flexibility [17]. Together, these findings provide a rationale for studying whether MDMA, when combined with psychotherapy, may enhance trauma-related processing while reducing avoidance and emotional overwhelm, especially during massed treatment in which therapy is completed over several weeks rather than several months.

While two Phase 3 trials of MDMA-assisted psychotherapy (MDMA-AT) have demonstrated large reductions in PTSD symptoms [18], [19], several limitations constrain interpretation and future clinical implementation. First, the lack of an active comparator and the presence of significant functional unmasking in these studies are major concerns, as 90% of all participants from the first Phase 3 trial correctly guessed treatment assignment [18], while 75% of participants in the control group and 94.2% of participants in the MDMA group in the second Phase 3 trial correctly guessed treatment assignment [19], [20], potentially inflating treatment effects [21]. Functional unmasking, combined with high treatment expectancy in these studies [22] and across MDMA and psychedelic studies more broadly, raises concerns about the ability to make causal inferences. Second, while the ‘psychotherapy only’ control arm of the Phase 3 trials demonstrated a large effect size for PTSD symptom reduction and had been manualized and refined over more than two decades [23], the ‘inner-directed therapy’ modality [24] was built around the MDMA experience and lacked prior evidence as a standalone treatment for PTSD (without MDMA) and had not been directly compared to standard PTSD therapies. Furthermore, the Phase 3 protocols involved three day-long dosing sessions and twelve non-dosing therapy sessions, requiring extensive therapist time and limiting scalability and accessibility. Finally, while one Phase 2 trial involved veteran participants outside of the VA [25], participants in the Phase 3 studies were primarily non-veteran, White, non-Hispanic women with a history of developmental trauma. The ability to generalize these findings to the veteran population seeking care within the VA healthcare system remains unclear.

In addition to the limitations of the Phase 3 trials, there is still uncertainty surrounding the most efficacious dosage of MDMA based on the wide range of dosages tested in previous MDMA trials overall, in which doses of 25 mg - 40 mg were used for active placebo comparator conditions vs. doses of 50 mg - 180 mg for the active therapeutic dose [26]. Using a range of possible MDMA doses as active comparators may therefore address the critical need for improved masking in MDMA trials and the need to explore different possible doses for efficacy. Three prior Phase 2 trials of MDMA-AT compared various doses of MDMA with the goal of improving masking; relative to the high level of correct guessing in the Phase 3 trials that included inactive placebo, accuracy was lower when different doses of MDMA were used [25], [27], [28]. The correct guess rate for participants in these trials ranged from 46% to 73%. While there is no accepted threshold for a successfully masked trial in the field of psychopharmacology, it has been suggested that a correct guess rate below 70% would indicate an adequately masked trial, a threshold that these low-dose controlled MDMA trials approached [20]. Rater masking also appeared to be potentially improved in these trials relative to the inactive placebo trials [20], suggesting that a truly double-masked MDMA study is possible through the use of low-dose controls.

Massed Exposure Therapy Enhanced with MDMA for PTSD (METEMP; [29]) is a novel model that integrates the ‘inner-directed therapy’ approach [23], [24] and prolonged exposure (PE) on dosing day, but uses a single MDMA dose paired with two weeks of massed exposure therapy completed during an intensive outpatient program. METEMP leverages translational evidence supporting MDMA's role in facilitating fear extinction [14] and utilizes an established, broadly disseminated, first-line PTSD therapy, PE, delivered in a massed format to capitalize on the putative “neuroplastic window” following MDMA administration. Preliminary open-label data from METEMP support further study, while requiring confirmation in controlled trials [30].

The present protocol, aMETEMP, is a version of METEMP that has been adapted for implementation in an outpatient integrative health care setting. The psychotherapy and integration are delivered via telehealth through multiple sessions per week over three weeks (instead of daily sessions over two weeks) in order to increase feasibility within the broader VA healthcare system. aMETEMP is designed to build on evidence supporting outpatient massed PE [31] and telehealth for the delivery of PTSD treatment within the VA [32]. aMETEMP has the potential to enhance efficiency, increase scalability and accessibility, reduce patient, provider, and system burden, and inform future research on whether and how MDMA-assisted therapy models could be evaluated within large healthcare systems, if supported by future evidence and regulatory decisions.

Importantly, our study design incorporates an incomplete disclosure strategy [33] in which participants are informed that they will receive a dose of MDMA anywhere between 35 mg and 125 mg. They are not told the specific number of arms in the trial or the specific dosages within this range that will be provided. This study design may inform future large-scale MDMA and psychedelic research by including active comparators, strategies to improve masking, and rigorous assessment of treatment expectation.

## 1 Materials and methods

### 1.1 Study design

This study is a randomized, double-masked pilot trial evaluating aMETEMP in up to 20 veterans in a VA outpatient healthcare setting. After giving informed consent and being deemed eligible for participation, veterans meeting DSM-5 criteria for PTSD [34] are randomized to receive a dose of MDMA (between 35 mg and 125 mg) during a single MDMA session integrated with a 3-week massed PE protocol that includes sessions to prepare for and process the MDMA sessions, and 12 exposure sessions (see Fig. 2). The primary aims are to evaluate feasibility, characterize safety/tolerability, assess masking integrity, and generate preliminary descriptive estimates of symptom change with assessments at baseline, post-treatment (1-3 weeks after the last PE session; primary endpoint), and 2- and 4-month follow-up. Randomization is conducted by the research pharmacy using concealed allocation procedures.

To mitigate attrition, we inform participants that they may be offered an open-label MDMA session and up to three additional integration sessions that would take place after their primary study treatment, depending on the dose to which they are initially randomized. At each participant's 2-month follow-up, they and their treatment providers are unmasked to the dose they were randomized to during treatment. This is done at the 2-month follow-up to ensure retention at the early pilot phase. Depending on the dose they previously received, they may be given the option to complete the additional sessions within 30 days of their 2-month follow-up.

### 1.2 Treatment expectancy and masking

#### 1.2.1 Expectancy

In clinical trials, participants’ beliefs and expectations about the intervention being investigated can impact how well they respond to the intervention; people believing that they will improve tend to have better outcomes than those who believe they will not improve [35], [36], [37]. While this phenomenon is not unique to MDMA-AT trials, the recent increase in media coverage promoting the effectiveness of psychedelic-assisted therapy makes assessing and controlling for treatment expectations especially important in these studies. Therefore, treatment expectancy is assessed using the Stanford Expectations of Treatment Scale (SETS; [38]) at three timepoints: baseline assessment, at the end of the MDMA preparation session, and at the start of the optional open-label MDMA session (if applicable).

#### 1.2.2 Masking

The possibility of confounding effects related to treatment expectations is increased if participants believe they know the treatment condition they are assigned to; therefore, it is crucial to take steps to prevent functional unmasking to treatment condition. In this study, participants, therapists, project coordinators, research assistants, and assessors are all masked to MDMA dose received during treatment, and participants are also masked to the number of possible arms and the exact dosages that are possible within those arms. Participants and study staff are only unmasked at each participant's 2-month follow-up assessment (not including the CAPS-5 assessors, who remain masked at each timepoint). Masking checks of both the participants and the therapists are administered at the end of the MDMA session, the post-treatment assessment, and the 2-month follow-up assessment.

To optimize rigor, mitigate expectancy bias, and improve integrity of masking compared to placebo-only designs [20], the study uses an incomplete disclosure strategy described in Matvey and colleagues [33]. Initially, 4 participants were informed of the specific MDMA doses that they could be randomized to; however, preliminary qualitative data revealed that participants' pre-existing beliefs about having a “profound psychedelic experience,” paired with knowledge of the potential treatment arms, were likely impacting their treatment expectations. Following the 4th participant's study completion, we shifted to using the incomplete disclosure strategy in an effort to better mitigate expectancy bias. Participants are now informed that they will receive a dose of MDMA that could be anywhere between 35 mg and 125 mg, but are not told the exact number of treatment arms nor the exact possible doses. This incomplete disclosure strategy decreases the amount of information about the study design provided to participants in order to decrease their ability to guess their assignment. This strategy also has the potential to reduce nocebo effects, as participants are all aware that they are receiving a dose of MDMA, rather than an “active” or “inactive” treatment, paired with a front-line evidence-based therapy for PTSD. In line with ethical standards for the use of incomplete disclosure, risks associated with each treatment arm are still disclosed during the informed consent process, and all participants undergo full debriefing at the 2-month follow-up. This includes disclosure of the study design and masking approach, and an opportunity to ask questions about their participation.

The actual MDMA dose each subject received will be compared to participant responses on the masking check questionnaire (see Fig. 1) to assess masking integrity qualitatively. It will also be compared with provider guesses as to treatment dose received, although providers are not masked to the trial design and so are aware of the exact potential doses the participants could have received.

**Fig. 1:**
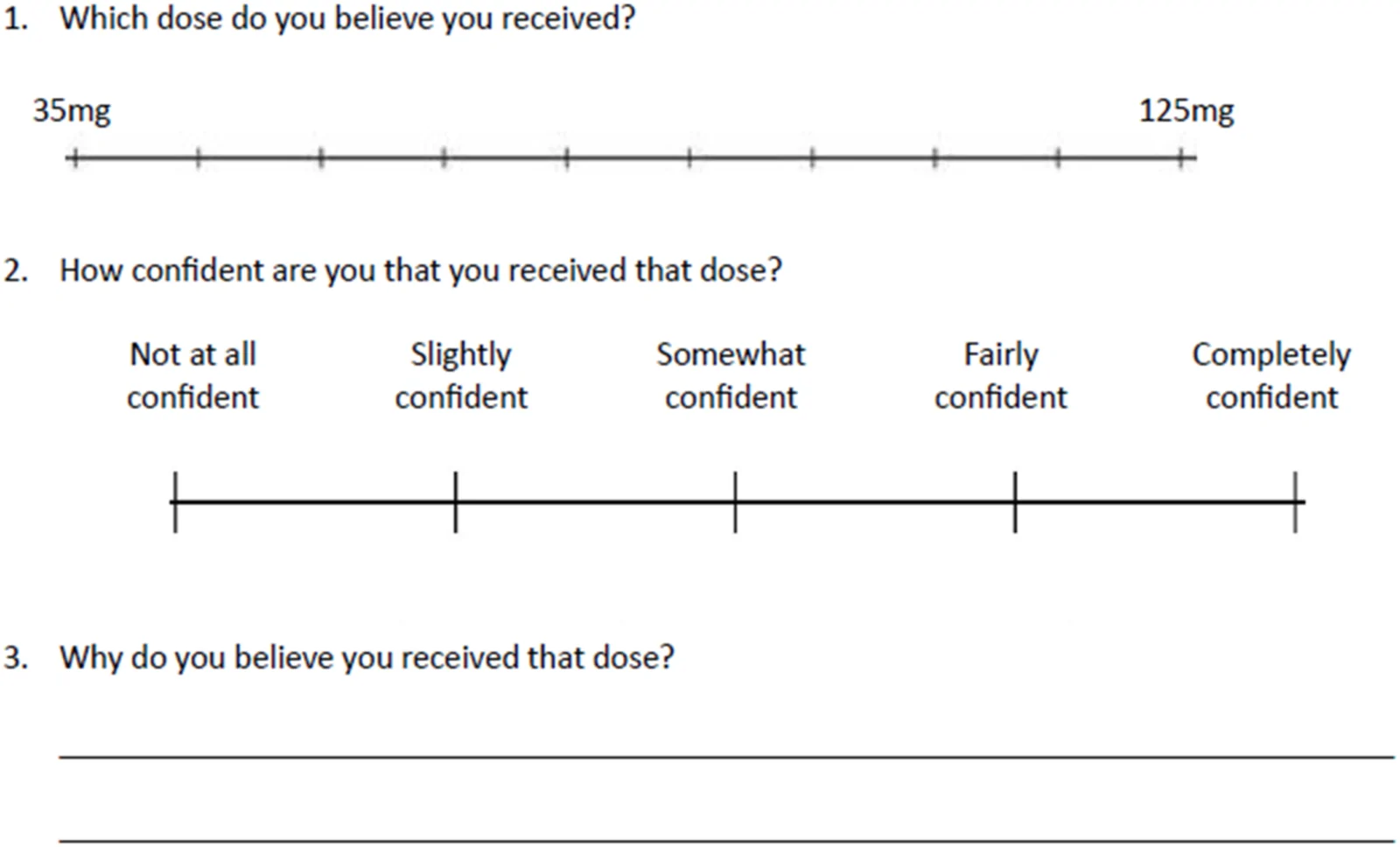
Participant masking assessment.

**Fig. 2:**
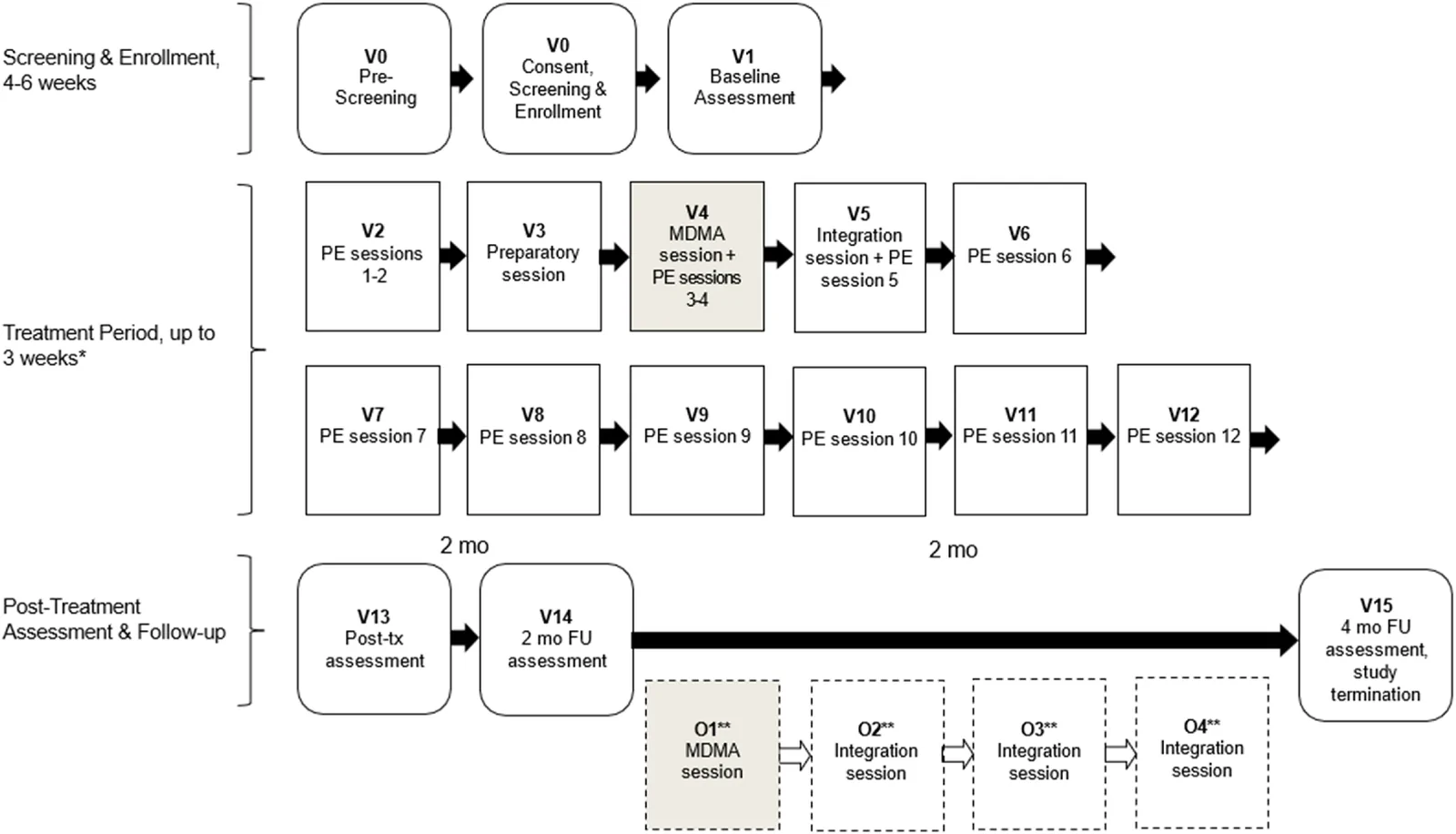
Study Timeline *Treatment period may be extended, if necessary, based on provider judgement **Optional, depending on randomized dose.

The masking checks were designed to collect information on participants' and providers’ beliefs about treatment without inadvertently reinforcing treatment expectations. Each masking check asks the participant/provider to make a guess about the dose received, their confidence in that guess on a 5-point Likert scale (“Not at all confident” to “Completely confident”), and their reasoning. The first masking check (see Fig. 1) is administered at the end of the MDMA session and asks participants to guess what dose they received on a scale from 35 mg to 125mg. This same masking check is administered to participants at their post-treatment assessment. The final masking check is done at the 2-month follow-up assessment. Participants are informed of the actual doses they could have received; with this additional information, we ask them to make a final guess about the dose they received along with their confidence in that guess, allowing us to capture a binary correct/incorrect datapoint. This strategy allows us to capture qualitative and quantitative masking data from participants and providers while not revealing the actual dose possibilities until after the primary assessment endpoint, minimizing the impact of treatment expectancy on outcomes.

### 1.3 Regulatory oversight

The study is being conducted under an FDA Investigational New Drug application. MDMA is a Schedule I investigational product and is not approved by FDA for the treatment of PTSD. The protocol was approved by the VA San Diego Healthcare System Institutional Review Board (#H230116; Initial Approval: 9/12/2023), VA Research and Development Committee, Research Advisory Panel of California (RAP-C), and Drug Enforcement Administration (DEA).

### 1.4 Participants

Participants are up to 20 veterans enrolled in the VA San Diego Healthcare System, aged 18–64, meeting DSM-5 PTSD criteria, and having a Clinician-Administered PTSD Scale for DSM-5 (CAPS-5) total severity score ≥ 25 [39]. Recruitment occurs through VA clinics, VA providers, self-referrals and medical chart review. Inclusion requires willingness to complete all study procedures, while exclusion criteria include psychiatric or medical contraindications to MDMA (see Table 1 and ClinicalTrials.gov NCT06117306 for a complete list of the eligibility criteria).

**Table 1:** Eligibility criteria.

Inclusion
Be a veteran (aged 18 - 64 years) with a primary diagnosis of PTSD based on clinical judgment from the study PI in consultation with the referring provider (if applicable) and meets criteria for PTSD on the CAPS-5, with a total severity score of ≥ 25.
Fluent in speaking and reading English
Willing to commit to medication dosing, therapy sessions, follow-up sessions, completing evaluation instruments, and all necessary telephone contact
Able to swallow pills
Agree to have study visits recorded, including the MDMA session(s), assessments, preparatory session, integration sessions, and PE sessions, and be aware that independent rater assessments for all sessions will occur
Have a contact able to drive the participant home after the MDMA session(s)
Provide a contact (relative, spouse, close friend, or other support person) who is willing and able to be reached by the investigators in the event of a participant becoming suicidal or unreachable
Have a personal contact (18+ years) who will stay with the participant overnight after they have returned home from the MDMA session and who is informed about the study and any possible risks to monitor for following the MDMA session
Agree to inform the investigators within 48 h of any new medical conditions or procedures
If able to become pregnant (i.e., assigned female at birth, fertile, following menarche and until becoming post-menopausal unless permanently sterile), must have a highly sensitive negative pregnancy test at study entry and prior to each MDMA session, and must agree to use adequate birth control through 10 days after the last MDMA session. Adequate birth control methods include intrauterine device (IUD), injected, implanted, intravaginal, or transdermal hormonal methods, abstinence, oral hormones plus a barrier contraception, vasectomized sole partner, or double barrier contraception. Two forms of contraception are required with any barrier method or oral hormones (i.e., condom plus diaphragm, condom or diaphragm plus spermicide, oral hormonal contraceptives plus spermicide or condom)
Agree to lifestyle modifications and comply with requirements for fasting and refraining from certain medications prior to MDMA sessions
Agree not to participate in other PTSD psychotherapy during study treatment
Not enroll in any other interventional clinical trials during the duration of the study, and commit to medication dosing, therapy, and study procedures
At pre-screening, have at least moderate PTSD symptoms in the last month based on PCL-5 total score of 40 or greater regarding a qualifying Criterion A trauma
May have well-controlled hypertension that has been successfully treated with anti- hypertensive medicines if they pass additional screening (as needed per study physician's clinical judgement) to rule out underlying cardiovascular disease
May have asymptomatic Hepatitis C virus (HCV) if it has previously undergone evaluation and treatment as needed
May have alcohol or substance use disorder if participant is not in withdrawal or requiring detox. Participants must have a plan, agreed upon by investigator, therapy team, and study physician, to reduce use of alcohol or other substances and to manage symptoms without self-medicating. Enrollment will require that, in the judgment of the investigator, therapy team, and study physician, the plan for decreasing substance use is realistic and has a good chance of succeeding to prevent substance use from impacting the safety or effectiveness of the investigational treatment
May have a history of or current Diabetes Mellitus (Type 2), if the condition is judged to be stable on effective management, and with approval by the study physician
May have hypothyroidism if taking adequate and stable thyroid replacement medication
May have a history of, or current, glaucoma if approval for study participation is received from an ophthalmologist

Exclusion

Not able to give adequate informed consent
Currently engaged in compensation and pension (C&P) litigation whereby financial gain would be achieved from prolonged symptoms of PTSD or any other psychiatric disorders
Likely, in the investigator's opinion and via assessment, to be re-exposed to their index trauma or other significant trauma, lack social support, or lack a stable living situation
Have used “ecstasy” (material represented as containing MDMA) or have participated in an MDMA clinical trial
Have any current problem which, in the opinion of the investigator or study physician, might interfere with participation due to it impacting the participant's safety and/or ability to participate in the protocol
Have hypersensitivity to any ingredient in MDMA HCl
Have received Electroconvulsive Therapy (ECT) within 12 weeks of enrollment
Have dementia
Have a history of or a current primary psychotic disorder assessed via the DIAMOND and clinical interview
Have a history of or current bipolar 1 disorder, bipolar 2 disorder, or manic episode assessed via the DIAMOND and clinical interview
Have a current eating disorder with active purging assessed via the DIAMOND and clinical interview
Have current major depressive disorder with psychotic features assessed via the DIAMOND
Have current panic disorder assessed via the DIAMOND
Have a history of amphetamine or cocaine substance use disorder
Have a current alcohol or substance use disorder other than caffeine or nicotine that the investigators, therapy team, and/or study physician judge to be a safety concern for enrollment in the study or that could interfere with the therapeutic process or with other aspects of study participation. Any participant who is not able to agree or adhere to a plan to reduce use and manage symptoms will not be enrolled.
Present with current serious suicide risk, as determined through psychiatric interview, responses to C-SSRS (scores of 4 or greater), and clinical judgment of the investigator; however, history of suicide attempts is not an exclusion. Any participant who is likely to require hospitalization related to suicidal ideation and behavior, in the judgment of the investigator, will not be enrolled. Any participant presenting with the following on the pre-screen C-SSRS will be excluded:
a. Suicidal ideation score of 4 or greater within the last 6 months of the assessment at a frequency of once a week or more b. Any suicidal behavior, including suicide attempts or preparatory acts, within the last 6 months of the assessment. Participants with non-suicidal self- injurious behavior may be included if approved by the study physician
Would present a serious risk to others as established through clinical interview and if necessary, discussion with treating psychiatrist
Require ongoing concomitant psychiatric pharmacotherapy, with the following exceptions permitted under the study protocol: opioid analgesics (limited to hydrocodone, morphine, and codeine), medications administered for the management of study-related AEs that are unresponsive to standard nonpharmacologic or pharmacologic interventions (e.g., benzodiazepines such as lorazepam for uncontrolled anxiety; sleep aids, excluding trazodone and prazosin), gabapentin when prescribed exclusively for pain management, stimulant medications prescribed for the treatment of ADHD, and cannabis use (provided it adheres to study-specific parameters and monitoring requirements). All psychoactive agents, herbal supplements, nonprescription products, and prescription medications will undergo comprehensive review by the research team to ensure compatibility with study requirements and participant safety.
Have a history of any medical condition that could make receiving a sympathomimetic drug harmful because of increases in blood pressure and heart rate. This includes, but is not limited to, a history of myocardial infarction, cerebrovascular accident, or aneurysm. Participants with other mild, stable chronic medical problems may be enrolled if the study physician and PI agree the condition would not significantly increase the risk of MDMA administration or be likely to produce significant symptoms during the study that could interfere with study participation or be confused with side effects of the MDMA. Examples of stable medical conditions that could be allowed include, but are not limited to Diabetes Mellitus (Type 2), Human Immunodeficiency Virus (HIV) infection, Gastroesophageal Reflux Disease (GERD), etc. Any medical disorder judged by the investigator to significantly increase the risk of MDMA administration by any mechanism would require exclusion.
Have a diagnosis of uncontrolled hypertension, defined by the American Heart Association as repeated readings of ≥ 140 mm of Mercury [mmHg] systolic or ≥ 90 mmHg diastolic, after treatment efforts
Have a history of ventricular arrhythmia at any time, other than occasional premature ventricular contractions (PVCs) in the absence of ischemic heart disease
Have Wolff-Parkinson-White syndrome or any other accessory pathway that has not been successfully eliminated by ablation
Have a history of arrhythmia, other than premature atrial contractions (PACs) or occasional PVCs in the absence of ischemic heart disease, within 12 months of screening. Participants with a history of atrial fibrillation, atrial tachycardia, atrial flutter or paroxysmal supraventricular tachycardia or any other arrhythmia associated with a bypass tract may be enrolled only if they have been successfully treated with ablation and have not had recurrent arrhythmia for at least one year off all antiarrhythmic drugs, as confirmed by a cardiologist
Have a marked Baseline prolongation of QT/QTc interval. For purposes of eligibility, this is defined as repeated demonstration of a QT interval corrected using Fridericia's formula [QTcF] >450 ms
Have a history of additional risk factors for Torsade de pointes (e.g., heart failure, hypokalemia, family history of Long QT Syndrome)
Require use of concomitant medications that prolong the QT/QTc interval during sessions
Have symptomatic liver disease or have significant liver enzyme elevations
Have history of hyponatremia or hyperthermia
Have a BMI of 18.5 or less
Are pregnant or nursing or are able to become pregnant and are not able/willing to practice an effective means of birth control
Have engaged in ketamine treatment or used ketamine within 12 weeks of enrollment
Have any preexisting conditions that can affect renal functioning

*Note.* PCL-5 = PTSD Checklist for DSM-5. CAPS-5 = Clinician Administered PTSD Scale for DSM-5. DIAMOND = Diagnostic Interview for Anxiety, Mood, and Obsessive Compulsive and Related Neuropsychiatric Disorders. C-SSRS = Columbia Suicide Severity Rating Scale.

### 1.5 Intervention

The aMETEMP intervention consists of 12 massed PE sessions (see Fig. 2; Table 3) delivered over approximately three weeks, all offered in person or virtually, except for PE sessions 3-4 (the MDMA session), which are in person. The initial session (PE sessions 1 & 2 combined into one 3.5-h visit) focuses on psychoeducation and development of a hierarchy list of in vivo exposures. The next visit is a 1.5-h MDMA preparatory session the business day prior to MDMA administration, in which participants are oriented to the MDMA session, provided with psychoeducation about MDMA, and given the space to ask questions about the MDMA session day. This psychoeducation emphasizes the variability in MDMA response, distinguishes MDMA from classic psychedelics, and tries to minimize specific expectations about the experience given that the subjective effects of MDMA can be influenced by a variety of biological, psychological, and environmental factors [12]. During this session, in addition to continuing to build therapeutic alliance and trust, the therapists provide guidance on what to expect during the MDMA session, and how to respond to the memories and feelings that could arise during treatment.

The business day after the preparation session is a 6-8 h in-person MDMA + PE session that includes alternating periods of inner-directed focus and interaction with the therapists. Consistent with the METEMP protocol, two guided imaginal exposures related to the pre-identified index trauma are completed during the MDMA session (PE sessions 3 & 4). The next day includes a 3-h session that consists of the integration session and PE session 5. The integration sessions are participant-led, unstructured periods where participants are invited to share their experiences from the MDMA session and any trauma-related insights that occurred during or since the MDMA session [23]. The remaining treatment visits are PE sessions 6-12. Consistent with traditional PE, imaginal exposures are conducted within these sessions and recorded, and participants complete assigned homework, including listening to recordings and engaging in vivo exposure assignments between sessions. Subjective Units of Distress are collected throughout sessions. Following the 2-month follow-up assessment, participants may be offered an optional additional MDMA session with up to 3 additional integration sessions, depending on the dose they received in their randomized session. PE sessions are delivered by a primary therapist, who is joined by a co-therapist for the preparation, MDMA, and integration sessions.

#### 1.5.1 MDMA sessions

The MDMA sessions are intended to support trauma processing and may yield trauma-related content that can be explored both within the session and in subsequent integration and PE sessions. MDMA sessions occur in a private room at the VA San Diego Healthcare System that has been specially designed for MDMA-assisted psychotherapy research [40]. This space is equipped with comfortable seating and access to nearby medical support if needed. The participant's urine drug toxicology screen and blood alcohol level are collected and confirmed to be negative the morning of the session. Temperature, blood pressure, and pulse are assessed and confirmed to be within protocol-defined acceptable ranges before, during, and at the end of the MDMA session, in consultation with a study physician who is on call for intervention if needed. Administration of MDMA occurs in the morning (9 a.m.) and the participant is required to stay on site (approx. 6-8hrs) and have planned transportation home with a prearranged driver after the session. The participant is encouraged to alternate between inner-directed exploration of emotional, cognitive, and somatic experiences [23], [24], optionally facilitated by eyeshades and curated music, and engagement with the therapists, which includes two imaginal exposures and subsequent processing. During the integration session that follows the day after the MDMA session(s) but also in subsequent PE sessions, participants may share insights or feelings related to their MDMA experience.

### 1.6 Therapist training and fidelity

Therapists are doctoral-level clinicians with PTSD treatment experience and training in PE and MDMA-AT. Training includes completion of MDMA-AT training through Resilient Pharmaceuticals and Sunstone Therapies, as applicable. Sessions are audio and video recorded and reviewed for fidelity, with supervision by expert massed PE clinicians and an expert MDMA therapist, producing independent adherence ratings.

### 1.7 Assessments

Participants are assessed at baseline, post-treatment, and 2- and 4-months after treatment. The primary clinical outcome is clinician-rated PTSD severity using the CAPS-5 [39]. Secondary clinical outcomes include self-reported PTSD symptoms (PCL-5; [41]), depression (PHQ-9; [42]), sleep (PSQI; [43]) and other psychosocial measures (see Table 2). Safety assessments include suicidality using the Columbia Suicide Severity Rating Scale (C-SSRS; [44]) and adverse event monitoring. Measures of treatment expectancy, satisfaction, and masking integrity are collected, along with qualitative interviews for additional feedback. Self-report surveys are completed in-person on paper or through VA REDCap surveys, depending on visit type and participant preference. See ClinicalTrials.gov NCT06117306 for all study measures and timepoints.

**Table 2:** Description of study measures.

Domain	Measure	Description and Psychometrics	Timepoint
Primary Clinical Outcome Measure			

Clinician-rated PTSD symptoms	Clinician-Administered PTSD Scale for DSM-5 (CAPS-5 [39])	30-item diagnostic interview assessing severity of four PTSD-related symptom clusters: re-experiencing, avoidance, negative alterations in cognition and mood, and alterations in arousal and reactivity.	Eligibility, All

Secondary Outcome Measures			

Self-reported PTSD symptoms	PTSD Checklist for DSM-5 (PCL-5 [41], [45])	20-item self-report questionnaire assessing presence and severity of PTSD symptoms. The participant rates their distress caused by PTSD symptoms on a 5-point Likert scale (1 = Not at all to 5 = Extremely).	Pre-eligibility, All, Every clinical visit
Psychosocial functioning	Brief Inventory of Psychosocial Functioning (B-IPF) [46]	7-item self-report questionnaire assessing functioning impairment across seven domains of intimate relationships, family and parenting, friendships, work, socializing, education, and self-care over the past 30 days.	All
Depressive symptoms	Patient Health Questionnaire-9 (PHQ-9) [42]	9-item self-report questionnaire assessing depressive symptoms with a score of 10 or above indicative of a possible major depressive episode. Higher total scores indicate greater severity of depressive symptoms.	All, Every clinical visit
Sleep	Pittsburgh Sleep Quality Index (PSQI) [43]	19-item self-report questionnaire assessing 7 subcategories of sleep quality over the last month. Participants respond using a combination of Likert scales (from 0 to 3) and open-ended questions that are later converted to scaled scores.	All
Sexual functioning	Changes in Sexual Functioning Questionnaire (CSFQ-14) [47]	14-item self-report survey assessing sexual functioning across five subscales: pleasure, desire/frequency, desire/interest, arousal/excitement, and orgasm/completion. All items are scored on 5-point scales with varying response options.	All
Moral injury	Moral Injury and Distress Scale (MIDS) [48]	Two-part self-report measure of potentially morally injurious events (PMIEs) exposure and moral injury symptoms in the past month. The first part of the scale uses 6 items to measure exposure to and bothersomeness of PMIEs through a 5-point Likert scale (0 = Not at all to 4 = Extremely). The second part includes open-ended questions to select and describe the most distressing PMIE followed by 18 items that are scored on 5-point Likert scales to indicate their cognitive, emotional, behavioral, social, and religious or spiritual reactions to their most distressing PMIE.	All
Adverse childhood events	Adverse Childhood Events Scale (ACES) [49]	10-item self-report survey assessing childhood stress. Participants indicate exposure to different kinds of abuse with yes/no responses, and answer with “1” if the abuse happened often.	Baseline
Posttraumatic growth	Posttraumatic Growth Inventory (PTGI) [50]	21-item self-report measure assessing level of posttraumatic growth across 5 subscales in adults who have experienced trauma. Participants respond using a 6-point Likert scale (1 = I did not experience this change as a result of my crisis, to 6 = I experienced this change to a very great degree as a result of my crisis).	All
Anger	State-Trait Anger Expression Inventory (STAXI-2) [51]	57-item self-report measure assessing experience, expression, and control of anger. Only the state anger (i.e., the intensity of anger at the time of completing the scale; 15 items) and trait anger (i.e., whether the person has an overall angry temperament; 10 items) scales used for this study, for a total of 25 items.	All
Well-being	Well-Being Signs (WBS) [52]	3-item self-report measure of psychosocial well-being. All items are rated on a scale from 0 (None of the time) to 10 (All of the time). An average score is calculated based on the ratings for each of the questions, with higher scores indicating better psychosocial well-being. Total scores range from 0 to 10.	All
Affect	Positive and Negative Affect Schedule (PANAS) [53]	20-item self-report measure of positive affect (PA) and negative affect (NA). All items are rated on a scale from 1 (Very Slightly or not at all) to 5 (Extremely). PA and NA items are summed separately with higher scores indicating more positive and negative affect respectively.	All, Visit 7

Safety Outcome Measures			

Suicidality	Columbia Suicide Severity Rating Scale (C-SSRS) [44]	Assesses suicidal ideation, ideation intensity, and behaviors during a clinical trial.	Pre-eligibility, All, Every clinical visit

Eligibility Measures			

Comorbid psychiatric disorders	Diagnostic Interview for Anxiety, Mood, and OCD and Related Neuropsychiatric Disorders (DIAMOND) [54]	60-min semi-structured clinical interview evaluating current and lifetime DSM-5 diagnoses.	Eligibility
Alcohol Use	Alcohol Use Disorders	10-item self-report test used to detect alcohol use disorders.	Pre-eligibility
	Identification Test (AUDIT) [55]		
Drug Use	Drug Use Disorders Identification Test (DUDIT [56])	11-item self-report measure used to detect substance use disorders.	Pre-eligibility
Trauma exposure	Life Events Checklist for DSM-5 (LEC-5) [57]	17-item self-report survey assessing exposure to potentially traumatic events.	Eligibility

Exploratory Measures			

Satisfaction with care	Client Satisfaction Questionnaire (CSQ-8) [58]	Satisfaction with healthcare and services received. Participants respond using a Likert scale from 0 to 3 with varying response options for each question.	Post-treatment
Acceptability of Care	Audiotaped Qualitative Interviews	Open ended questions assessing general acceptability of participant experience.	Post-treatment, 2-months, 4-months post-treatment
Treatment expectations	Stanford Expectations of Treatment Scale (SETS) [38]	10-item self-report questionnaire that assesses a patient's expectations about their upcoming treatment.	Baseline, Preparation, Optional Additional Medicine Session

*Note.***“**All” includes the four assessment timepoints: baseline, post-treatment, 2-months post-treatment, and 4-months post-treatment. The CAPS-5 is administered for eligibility during the Screening visit, and if there is no medication taper and/or delay of more than 30 days between the Screening visit and first treatment visit, then it will be used as the Baseline CAPS-5.

**Table 3:** Description of study treatment.

Visit #	Session Content	Session Length	# Therapists
2	PE Sessions 1 + 2: psychoeducation and development of a hierarchy list of in vivo exposures	∼3.5 h	1 (Primary)
3	Preparation Session: orientation to MDMA session, psychoeducation about MDMA, and addressing any questions	∼1.5 h	2 (Primary + Co-Therapist)
4	MDMA Session + PE Sessions 3 + 4: MDMA administration followed by alternating periods of inner-directed focus and interaction with the therapists as well as two guided imaginal exposures	∼6-8 h	2 (Primary + Co-Therapist)
5	Integration Session + PE Session 5: participants share their experiences from the MDMA session and any trauma-related insights as well as a guided imaginal exposure	∼3 h	2 (Primary + Co-Therapist) during integration, 1 (Primary) during PE session 5
6-12	PE Sessions 6-12: guided imaginal exposures are completed in-session, with in vivo exposures assigned to be completed between sessions	∼1.5 h each	1 (Primary)

*Note*. Visit 1 is the baseline assessment and not a part of the study treatment.

### 1.8 Analysis

The sample size of up to 20 participants is intended to evaluate feasibility and acceptability, characterize safety/tolerability, assess masking integrity, and generate preliminary descriptive estimates to inform a future randomized controlled trial. This preliminary study is not powered to detect statistically significant effects or differences between MDMA groups, so only descriptive statistics will be reported for primary and secondary outcomes without tests of within-subjects or between-group differences. Missing data will be handled using full information maximum likelihood (FIML) analyses and/or multiple imputation prior to analysis. Preliminary efficacy will be derived primarily from descriptive statistics for within-person change in primary and secondary outcomes from baseline to post-treatment, as well as maintenance of change from post-treatment through 2- and 4-month follow-up. Effect sizes will be calculated based on growth curve estimated slopes and/or model-implied estimated means and standard deviations at each assessment, using standardized mean change for within-group change (i.e., pooled standard deviations and a repeated-measures correction). Exploratory growth-curve analyses may be used to estimate the magnitude and trajectory of symptom change over time; these analyses will be interpreted descriptively given the pilot sample size. Nonlinear (e.g., quadratic or piecewise) slope parameters will be tested if appropriate based on visual inspection of the data.

Main analyses for the primary outcome (change in PTSD symptom total severity score assessed by CAPS-5) will compare dose groups using the modified intent-to-treat (mITT) sample of veteran participants who completed the MDMA session and had at least one follow-up CAPS-5 assessment. Analysis will be conducted following the final 4-month assessment at the conclusion of the study. Reliable and clinically significant change will be calculated for each participant using the method described by Jacobson and Truax [59], which uses sample-specific dispersion parameters and is considered best practice in clinical psychology. Sensitivity analyses will be conducted using the sample of participants who completed treatment.

Main and sensitivity analyses for all exploratory secondary outcomes (see Table 2) will use the same methods as the primary outcome. Additionally, moderation analyses will explore whether the presence of secondary traumatic stressors (as assessed by the Life Events Checklist for DSM-5 [57]) and/or adverse childhood experiences (as assessed by the Adverse Childhood Experiences [49] scale) moderate the degree of improvement in CAPS-5 PTSD severity scores during the study.

## 2 Discussion

Veterans with PTSD continue to face high rates of non-remission and dropout from first-line therapies. While MDMA-AT holds promise, trials to date have been limited by functional unmasking, lack of active comparators, and protocols that are difficult to scale. aMETEMP is designed to address several of these limitations. We adapted the previously developed METEMP model into a practical outpatient protocol for the Veterans Health Administration (VHA) that combines a single MDMA dose with three weeks of telehealth or in-person delivered massed PE, a gold standard PTSD therapy. Given that 90–94% of participants in Phase 3 MDMA-AT trials correctly guessed treatment assignment, this protocol uses a range of MDMA doses plus an incomplete disclosure strategy to improve masking. This approach has already shown improved masking in prior dose-response MDMA-AT trials and may improve interpretability relative to designs using inert placebo, although causal conclusions will remain limited by the pilot sample size and study design.

Designed for real-world VHA implementation, aMETEMP sessions are primarily delivered via telehealth, reducing burden and increasing access. Our goal is to generate data that will inform future large scale clinical trials designed to address key concerns about MDMA-AT raised by the FDA [60] and VA [61], [62] regarding masking, scalability, and using an evidence-based trauma-focused therapy approach. Notably, the protocol's use of a range of MDMA doses as active comparators, coupled with an incomplete disclosure strategy, represents a methodological advancement to address the persistent issue of functional unmasking across psychedelic research. This design aims to enhance the credibility of the masking process, as preliminary findings from prior studies suggest that masking is more effective when using lower doses of MDMA comparators rather than inert placebo [20], [25], [27], [28]. Building on these findings, our protocol integrates comprehensive psychoeducation and an intentional incomplete disclosure approach to further minimize expectancy effects and better characterize treatment effects and expectancy-related influences in future research.

A potential critique of this approach is that lower doses of MDMA may still provide some therapeutic benefit for PTSD. This pilot may provide preliminary descriptive signals regarding symptom change, tolerability, subjective experience, and masking across different MDMA doses, inform optimal selections for both active and control conditions, assess subjective experiences related to treatment response and unmasking, and evaluate strategies to mitigate expectancy effects. Additionally, these data will help refine trial design elements important for regulatory and clinical acceptance, potentially informing the design of future trials evaluating MDMA as an augmentation strategy for evidence-based PTSD psychotherapy.

This pilot protocol has several limitations. The study is not powered to detect statistically significant within- or between-dose effects, and efficacy-related findings will be interpreted descriptively. The disclosure strategy was modified after the first four participants, which may affect expectancy and masking outcomes. Accordingly, masking and expectancy findings will be interpreted in light of potential differences between participants enrolled before and after this change. Although active-dose comparators and incomplete disclosure are intended to improve masking, functional unmasking and expectancy effects may still influence participant and clinician-reported outcomes. Finally, the sample is limited to veterans receiving care within one VA healthcare system, which may limit generalizability. Similarly, a limitation of this study and other MDMA trials is the set of exclusion criteria, which limit participation to only some veterans with PTSD, e.g., those who have not been suicidal in the past 6 months and who are able to safely taper off medications. Further studies should be designed to determine whether more relaxed exclusion criteria could be implemented safely, including research specifically investigating the interaction of MDMA with other psychiatric medications.

Given the use of incomplete disclosure as a masking strategy, we recognize the ethical imperative to prioritize participant autonomy and informed choice throughout the protocol. Due to the consciousness-altering effects of these substances, ensuring ethically appropriate informed consent is especially salient in research involving MDMA or other psychedelics, particularly when an IRB-approved incomplete disclosure strategy is used. To address this, the aMETEMP protocol emphasizes clear communication about the voluntary nature of all exposure-related activities and ensures that participants retain control over the pacing, intensity, and continuation of such interventions. Study therapists are trained to continually assess participants’ comfort and willingness to proceed, providing multiple opportunities to reaffirm consent before and during dosing sessions. By reinforcing participant agency in this manner, we aim to balance methodological rigor in masking with respect for the autonomy, rights, and well-being of our participants.

Overall, the aMETEMP model is designed with the unique needs of large health care systems like VA in mind, including feasibility and scaling, should FDA approve an MDMA-containing therapy for the treatment of PTSD in the future. The majority of participant visits are virtual, and the use of telehealth is a novel contribution that increases implementation feasibility of the protocol. Findings from this pilot may inform the design of future multi-site trials and, if supported by future evidence and regulatory decisions, implementation considerations.

## CRediT authorship contribution statement

**L.A. Morland:** Writing – review & editing, Writing – original draft, Supervision, Methodology, Investigation, Funding acquisition, Conceptualization. **B. Chargin:** Writing – review & editing, Writing – original draft, Project administration, Methodology, Investigation, Data curation. **B. Rothbaum:** Writing – review & editing, Writing – original draft, Methodology, Investigation, Conceptualization. **J.L. Maples-Keller:** Writing – review & editing, Writing – original draft, Methodology, Investigation, Conceptualization. **T. Wachsman:** Writing – review & editing, Project administration, Methodology, Investigation, Data curation. **S.B. Norman:** Writing – review & editing, Methodology, Investigation. **S.N. Adams:** Writing – review & editing, Methodology, Investigation. **D. Perivoliotis:** Writing – review & editing, Methodology, Investigation. **A. Alam:** Writing – review & editing, Supervision, Methodology, Investigation. **C.S. Stauffer:** Writing – review & editing, Supervision, Methodology, Investigation. **A.C. Angkaw:** Writing – review & editing, Methodology, Investigation. **V. Marquez:** Writing – review & editing, Methodology, Investigation. **J. Woolley:** Writing – review & editing, Writing – original draft, Methodology, Investigation, Conceptualization. **J.M. Kopelman:** Writing – review & editing, Writing – original draft, Methodology, Investigation, Conceptualization. **P.P. Schnurr:** Writing – review & editing, Writing – original draft, Resources, Methodology, Investigation, Funding acquisition, Conceptualization.

## Ethics declaration

Written informed consent to take part in the study and to publish the article has been obtained from all participants or their legal representatives. The privacy rights of participants have been observed.

This study was performed in compliance with relevant laws, regulatory frameworks and guidelines where the research took place. This study was approved by the VASDHS Institutional Review Board(Approval No. H230116).

The results of this clinical trial and any associated work have been posted in a registry. This clinical trial was registered with number NCT06117306.

## Declaration

**No** generative AI tools were used in the manuscript preparation.

## Funding source

This work was supported by Healing Breakthrough, a program of the Heroic Hearts Project (#10285). JMK is supported by NIMH through the R25 UCSD Psychiatry Residency Research Track (MH101072). The funding source had no role in study design, data collection, analysis, or manuscript preparation.

## Declaration of competing interest

The authors declare the following financial interests/personal relationships which may be considered as potential competing interests:Leslie Morland reports financial support was provided by Healing Breakthrough. Paula Schnurr reports financial support was provided by Healing Breakthrough. Christopher Stauffer reports a relationship with Steven & Alexandra Cohen Foundation that includes: funding grants. Christopher Stauffer reports a relationship with Dr. Bronner Family Foundation that includes: funding grants. Barbara Rothbaum reports a relationship with Wounded Warrior Project that includes: funding grants. Barbara Rothbaum reports a relationship with National Institutes of Health (NIH) that includes: funding grants. Barbara Rothbaum reports a relationship with U.S. Department of Defense (DoD) that includes: funding grants. Barbara Rothbaum reports a relationship with National Science Foundation (NSF) that includes: funding grants. Barbara Rothbaum reports a relationship with Bob Woodruff Foundation that includes: funding grants. Barbara Rothbaum reports a relationship with Multidisciplinary Association for Psychedelic Studies (MAPS) that includes: funding grants. Barbara Rothbaum reports a relationship with Virtually Better that includes: equity or stocks. Barbara Rothbaum reports relationships with Otsuka, Psychwire, Senseye, MAPS, Lykos, Jazz Pharmaceuticals, GoodCap, Transcend, and Penumbra that includes: consulting or advisory. Barbara Rothbaum reports a relationship with Anxiety and Depression Association of America (ADAA) that includes: board membership. Barbara Rothbaum reports a relationship with National Center for PTSD that includes: board membership. Barbara Rothbaum reports a relationship with GratitudeAmerica that includes: board membership. Barbara Rothbaum receives royalties from Oxford University Press, American Psychiatric Association Publishing, and Emory University. Paula Schnurr received royalties from Guilford Press. If there are other authors, they declare that they have no known competing financial interests or personal relationships that could have appeared to influence the work reported in this paper.

## Data Availability

No data was used for the research described in the article.
